# Computational Modelling of a Prestressed Tensegrity Core in a Sandwich Panel

**DOI:** 10.3390/ma18214880

**Published:** 2025-10-24

**Authors:** Jan Pełczyński, Kamila Martyniuk-Sienkiewicz

**Affiliations:** Faculty of Civil Engineering, Warsaw University of Technology, Al. Armii Ludowej 16, 00-637 Warsaw, Poland; kamila.martyniuk-sienkiewicz.dokt@pw.edu.pl

**Keywords:** tensegrity, finite element modelling, prestress, sandwich panels

## Abstract

Tensegrity structures, by definition composed of compressed members suspended in a network of tensile cables, are characterised by a high strength-to-weight ratio and the ability to undergo reversible deformations. Their application as cores of sandwich panels represents an innovative approach to lightweight design, enabling the regulation of mechanical properties while reducing material consumption. This study presents a finite element modelling procedure that combines analytical determination of prestress using singular value decomposition with implementation in the ABAQUS™ 2019 software. Geometry generation and prestress definitions were automated with Python 3 scripts, while algebraic analysis of individual modules was performed in Wolfram Mathematica. Two models were investigated: M1, composed of four identical modules, and M2, composed of four modules arranged in two mirrored pairs. Model M1 exhibited a linear elastic response with a constant global stiffness of 13.9 kN/mm, stable regardless of the prestress level. Model M2 showed nonlinear hardening behaviour with variable stiffness ranging from 0.135 to 1.1 kN/mm and required prestress to ensure static stability. Eigenvalue analysis confirmed the full stability of M1 and the increase in stability of M2 upon the introduction of prestress. The proposed method enables precise control of prestress distribution, which is crucial for the stability and stiffness of tensegrity structures. The M2 configuration, due to its sensitivity to prestress and variable stiffness, is particularly promising as an adaptive sandwich panel core in morphing structures, adaptive building systems, and deployable constructions.

## 1. Introduction

Tensegrity structures represent an architectural and engineering paradigm in which isolated compression members (struts) are suspended in a continuous tension network (cables), creating self-equilibrating systems that achieve structural stability through prestress. As discussed by [1,2], this principle enables the formation of mechanically efficient, self-stable systems in which equilibrium is achieved through internal force balance rather than rigid connections. This unique structural philosophy, characterised by discontinuous compression and continuous tension, offers exceptional strength-to-weight ratios, For instance, Wang et al. [3], Feng et al. [4], and Xue et al. [5] demonstrated that tensegrity structures can achieve material-efficient designs in both rigid and soft structures, allowing significant reductions in mass without compromising performance. It also provides inherent flexibility, enabling controlled deformation under load without permanent damage, as observed in previous experimental studies [1,6,7], and the capacity for large, recoverable deformations [1,5]. The integration of tensegrity principles into sandwich panel cores presents a novel approach to lightweight structural design, enhancing their adaptability and mechanical performance while reducing material consumption. Recent studies indicate that tensegrity and related lattice architectures enable high stiffness-to-weight ratios and material-efficient layouts, which is advantageous for applications requiring low mass and high structural performance [3,8].

The significance of these features extends far beyond academic exploration. Tensegrity-based sandwich panels hold promise for deployable space structures—where rapid deployment and reconfigurability are critical [9,10]—as well as adaptive building systems [5,7] and next-generation composite materials [7]. For example, Shang et al. [9] presented tensegrity-based deployable units for aerospace applications, whereas Al Sabouni-Zawadzka et al. [7] and Xue et al. [5] explored their morphing behaviour in adaptive materials. Unlike conventional sandwich panels, which typically employ solid or foam cores that provide lightweight support but lack adaptive behaviour, tensegrity cores inherently enable tunable stiffness through prestress adjustment [11], exhibit energy absorption capabilities [12,13], and provide pathways for self-deployment in demanding environments. These advantages position tensegrity as a transformative concept in structural and material engineering, particularly in contexts where efficiency, resilience, and adaptability are paramount. Recent advances in computational modelling and manufacturing technologies have enabled the practical implementation of tensegrity structures in civil engineering applications [14,15,16]. These works show that both accurate numerical modelling and advanced fabrication techniques are necessary to translate tensegrity concepts into functional structures.

**Figure 1:**
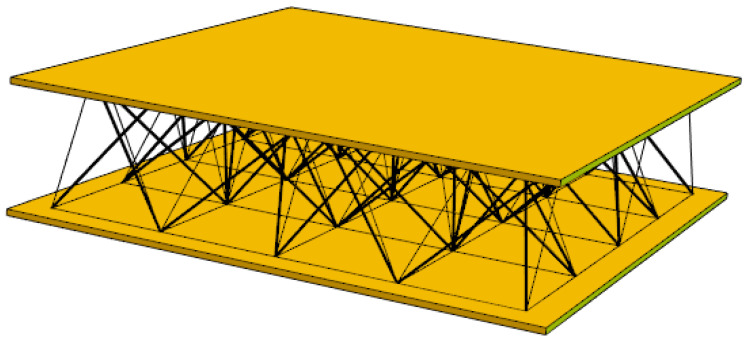
Schematic representation of a sandwich panel with a tensegrity core. The structure consists of two rigid facesheets connected by a three-dimensional network of struts and cables forming a tensegrity lattice.

In the context of this study, it is assumed that facesheets may be manufactured from standard engineered wood products, such as oriented strand boards (OSBs), which emphasises the need for the core to remain lightweight. To clearly illustrate this design philosophy, a scheme is proposed in Figure 1, where two thin facesheets are connected by a tensegrity lattice, highlighting the distinction between conventional passive cores and the adaptive tensegrity solution.

To fully exploit these unique mechanical features in real applications, robust numerical tools are required to capture their nonlinear behaviour. Finite element analysis of tensegrity structures presents significant computational challenges due to their geometrically nonlinear response and the critical importance of accurate prestress implementation [17]. The structural behaviour of these systems is highly sensitive to initial stress states, member geometry, and boundary conditions [18]. Moreover, the integration of multiple tensegrity modules into larger structural assemblies introduces additional complexities related to load transfer and global stability [3,17,18].

The development of accurate computational models for prestressed tensegrity cores requires sophisticated computational strategies capable of handling singularities in the unstressed state, implementing precise prestress distributions, and capturing the characteristic nonlinear load–deformation relationships of these systems [19,20]. The present study addresses these challenges by introducing a comprehensive finite element modelling framework that combines analytical prestress determination through singular value decomposition [21,22] with implementation in commercial finite element software. Custom Python 3 tools were developed for automatic generation of geometry, element sets, and prestress definitions; Wolfram Mathematica provided the algebraic foundation for single-module analysis, while ABAQUS™ 2019 facilitated the simulation of multi-module structures. This division of tasks between dedicated mathematical software and commercial FEA ensured both computational flexibility and high accuracy in tensegrity modelling.

Several studies have addressed the stability (both prestress-induced and buckling), geometric nonlinearity, and practical design of tensegrity lattices [23,24,25]. These works provide useful insights into tensegrity structures in general, yet they do not directly consider tensegrity-inspired cores for sandwich panels.

Despite these promising attributes, the literature lacks systematic computational studies of tensegrity cores specifically designed as sandwich panel components. In particular, it is necessary to clarify how different module arrangements influence stiffness, stability, and sensitivity to prestress. Addressing this gap, the present study investigates two representative multi-module tensegrity configurations (M1 and M2) in order to assess their mechanical behaviour under prestress and external loading. The comparative analysis of these models provides insight into the role of modular composition in shaping global stiffness and stability, thus supporting the rational design of tensegrity-based sandwich panels for engineering applications.

This paper is organised as follows. Section 2 specifies the materials and methods applied in this study, including the description of basic tensegrity equations and the procedure of modelling single and multi-module tensegrity structures in ABAQUS™ 2019 software. Section 3 reports the results of the computational analysis performed on two multi-module tensegrity structures. The conclusions of the present study and detailed discussion are given in Section 4.

## 2. Materials and Methods

### 2.1. Finite Element Modelling of a Singe Tensegrity Module

From a mechanical perspective, tensegrity systems can be analysed as truss-like structures and represented by discrete models. Two principal approaches are used for this purpose: the finite element method (FEM) [26] and the direct algebraic formulation [27,28,29]. Both are equally rigorous and capture the influence of prestress on the global response of the system. The FEM follows a conventional and well-structured procedure: evaluation of single elements, assembly of global stiffness matrices, and application of boundary conditions. The algebraic method, in contrast, is mathematically more compact, but requires that the global matrices be established directly at the start of the analysis. The formal equivalence of the two strategies was demonstrated by [28].

In the present study, an ideal truss model is adopted, in which all members are connected by perfect pin joints and transmit only axial forces. In the joints, no bending stiffness or transverse shear transfer is considered. This idealisation allows for a clear evaluation of the influence of prestress on the stiffness and stability of the tensegrity core but also makes the system highly sensitive to any geometric or numerical imperfections. As a consequence, even small coordinate perturbations can lead to the loss of equilibrium, which limits the possibility of introducing imperfections directly in the current formulation.

According to [28] for planar tensegrity and later generalised by [29] to spatial cases, the governing equations can be written in the standard algebraic form

(1) Kq=Q,

with the stiffness matrix expressed as

(2) $$K=B^{⊺}EB+C^{⊺}SC+{\hat{C}}^{⊺}S\hat{C},$$

where q and Q denote nodal displacements and nodal force vectors, respectively. The matrices E and S are diagonal and defined as

(3) $$E=\mathrm{diag}\left(\frac{EA_{K}}{L_{K}}\right),\;S=\mathrm{diag}\left(\frac{S_{K}}{L_{K}}\right).$$

Here, $EA_{K}$ is the axial stiffness, $L_{K}$ the length of the member, and $S_{K}$ the prestress in the *K*-th bar. The matrices B, C, and $\hat{C}$ are geometry-dependent and link the local and global coordinate systems. Their derivation is well documented in earlier work [27,28,29].

**Figure 2:**
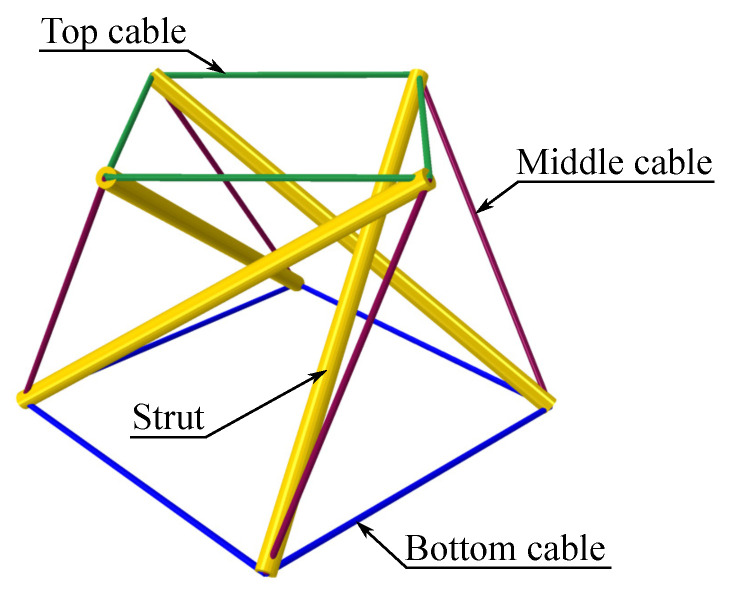
Four-strut *simplex* tensegrity module.

The prestress distribution, that is, the set of equilibrated forces $S_{K}$, is obtained from the singular value decomposition (SVD) of the matrix B [7,28,29]. This decomposition allows one to distinguish tension and compression members, thus identifying cables and struts within the tensegrity module (see Figure 2).

In ABAQUS™ 2019 modelling, the correct representation of geometry and prestress is critical. The reason is that tensegrity structures inherently possess infinitesimal rigid body mechanisms, which make the stiffness matrix initially singular and highly sensitive to the assumed stress state. For this reason, exact values of prestress and member geometry must be provided.

**Figure 3:**
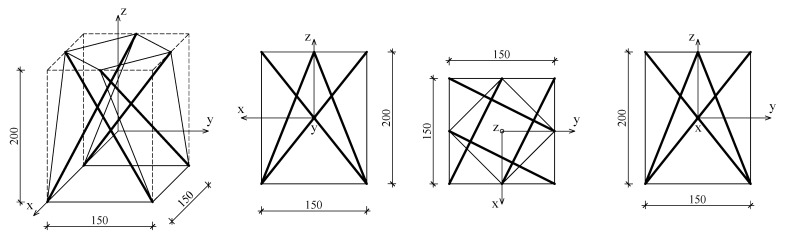
Dimensions of the four-strut *simplex* tensegrity module used in calculations. Thicker lines indicate struts, thin lines indicate cables. Dimensions are given in milimeters.

The proposed method for implementing the FEM model is as follows. First, a Wolfram Mathematica script is used to derive the extension matrix B of a single *simplex* module with four struts, with dimensions shown in Figure 3. This is necessary to determine the prestress state, which will be introduced later in the FEM model. The node coordinates are then written into the *Part section of the ABAQUS™ 2019 input file. The structure is discretised with 2-node truss elements (T3D2) [30], grouped into four sets: struts, top_cables, bottom_cables and middle_cables, according to Figure 2. Each cable in the tensegrity module was modelled as an individual truss element connected to the corresponding nodes at its ends. The connections between the cables and the struts were assumed to be ideal pin joints, preventing any relative sliding along the struts. Consequently, the continuous cables visible in the physical configuration were represented by sets of separate elements that met at common nodes. The prestress introduction procedure did not modify the geometry or element lengths; therefore, the initial configuration of the cable network remained fixed during subsequent deformation.

The node coordinates are as follows:
*Part, name=Part-1*Node1,150.,0.,0.2,150.,150.,0.3,0.,150.,0.4,0.,0.,0.5,75.,0.,200.6,0.,75.,200.7,150.,75.,200.8,75.,150.,200.
Each group received its own *Solid Section definition, specifying the cross-sectional area.
*Solid Section, elset=struts_sec, material=Steel112.723,*Solid Section, elset=cables_sec, material=Steel2.54469,
where the sets of elements struts_sec and cables_sec contain all struts and cables, respectively. In this example, it is assumed that the struts are made of elements with a circular cross-section with a radius $r_{s}=2.01$ mm and cables with a circular cross-section with a radius $r_{c}=0.90$ mm. For such a simplex module, the prestress values (dependent on the scale factor $S_{0}$, given in newtons) are given in Table 1.

The prestress can then be introduced via the *initial conditions option, placed between the *Assembly and *Step blocks. This allows the previously determined prestress forces to be directly imposed:
*initial conditions, type=stressstruts,  −2.8379566311743187top_cables,  5.766321129528219bottom_cables,  4.077392451947111middle_cables,  11.612455515703262
This procedure guarantees precise control of prestress levels. Alternatively, one may specify the prestress in a single bar, letting ABAQUS™ 2019 redistribute the forces, but in practice this option gives less control over individual member stresses.

From a practical perspective, the tensegrity modules maintain the relative ratios of member forces, which allows for external verification of the prestress state. In a physical prototype, the tension in accessible cables can be measured directly, providing a means to ensure that the desired prestress distribution has been achieved. Mechanically, a turnbuckle procedure represents the simplest approach to impose and adjust the cable forces at the scale considered. Active control of cable tension could be considered in more complex systems, for example, when simultaneous adjustment of multiple diagonal or base cables is required. However, such an approach introduces additional complexity due to the need for actuators and control hardware. These practical aspects are indicative and suggest directions for future experimental validation of the proposed tensegrity core systems.

The analysis is then performed in two nonlinear static steps:
*Step, name=Step-1, nlgeom=YES*Static1., 1., 1e-05, 1.** …*End Step***Step, name=Step-2, nlgeom=YES*Static1., 1., 1e-05, 0.02** …*End Step
The first step (Step-1) is essential to stabilise the prestress field and remove singularities of the stiffness matrix. Only in the second step (Step-2) are the external loads applied, which yields the actual response of the system.

A key feature of the adopted procedure is the manual definition of prestress forces, which provides full control of the prestress distribution. In contrast to the automatic initial stress calculation of ABAQUS™ 2019, this approach reproduces the results of the SVD analysis of matrix B with high precision.

### 2.2. Structures Composed of Multiple Tensegrity Modules

**Figure 4:**
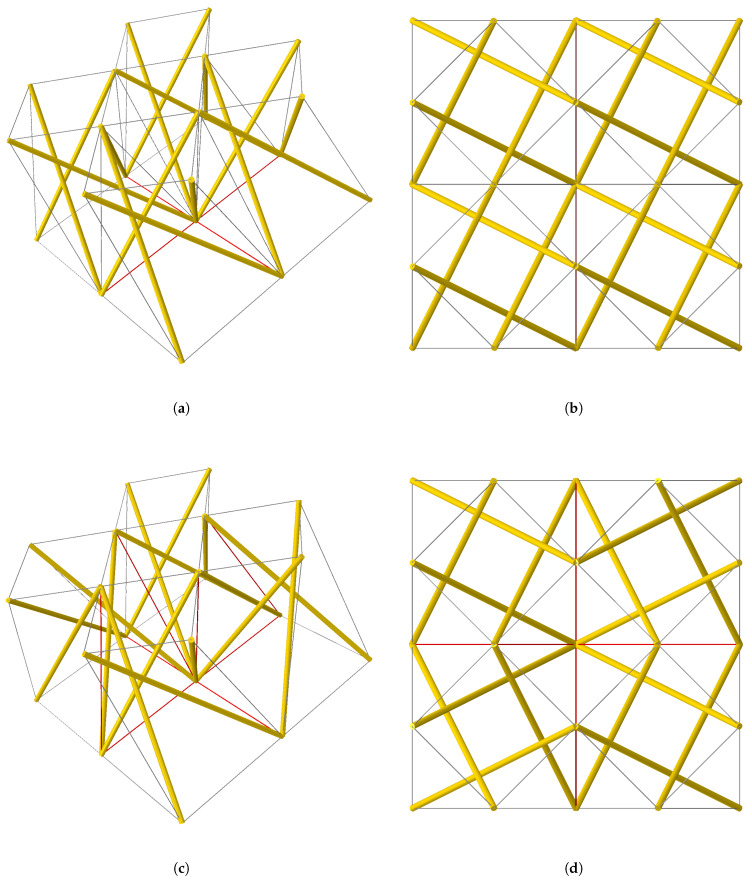
Two structures consisting of four tensegrity modules connected at adjacent nodes: (**a**,**b**) show the M1 structure formed by four identical modules, its axonometric projection, and top view, respectively; (**c**,**d**) show the M2 structure consisting of two modules in their original configuration and two mirror-image modules, its axonometric projection and top view, respectively. The colours indicate the following: grey—single cables, red—duplicate cables, yellow—struts.

Model M1, illustrated in Figure 4a,b, is composed of four identical tensegrity modules, shown in Figure 3, connected at adjacent nodes. In this configuration, all modules maintain the same handedness. In addition, the eight cables located at the base (highlighted in red in Figure 4a) overlap in pairs.

Model M2, shown in Figure 4c,d, is also composed of four tensegrity modules connected at adjacent nodes. In this case, two modules preserve their original handedness, whereas the other two are mirrored with respect to a vertical plane. As a result, in addition to the eight overlapping base cables, eight other middle cables (marked red in Figure 4c) also overlap in pairs.

**Figure 5:**
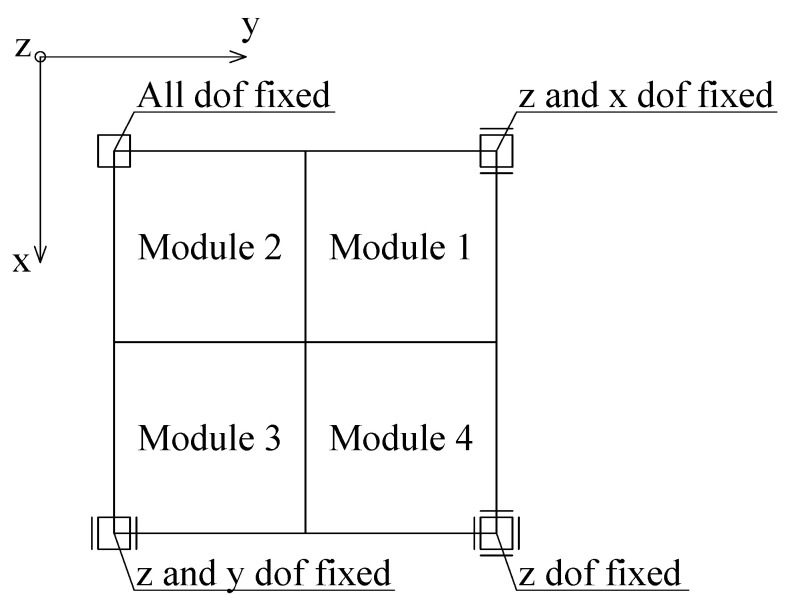
Support conditions for models M1 and M2, top view.

In both models, identical boundary conditions were applied at the base nodes. At one of the corner nodes, all three translational degrees of freedom were restrained. At the opposite corner node, only the vertical displacement was fixed. At the two remaining corners, the vertical displacement was restrained together with one horizontal displacement at each node, in mutually perpendicular directions. The support conditions are schematically illustrated in Figure 5.

In each model, two loading scenarios were considered. In the first scenario, four vertical downward concentrated forces were applied to the four internal nodes of the top plane of the structure, each with a magnitude of 2.5 N. In the second scenario, a vertical downward displacement of 5 mm was imposed at all nodes of the top plane of the model. ABAQUS™ 2019 input files with exemplary data are available at https://doi.org/10.5281/zenodo.17132705.

## 3. Results

**Figure 6:**
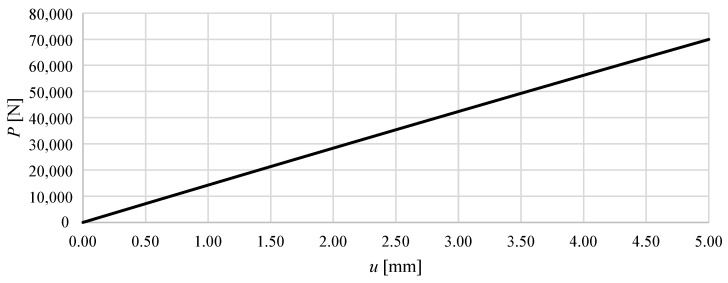
The total vertical reaction *P* as a function of the imposed displacement of the upper nodes of the structure *u* for Model M1, assuming a prestress level of $S_{0}=100$ N.

**Figure 7:**
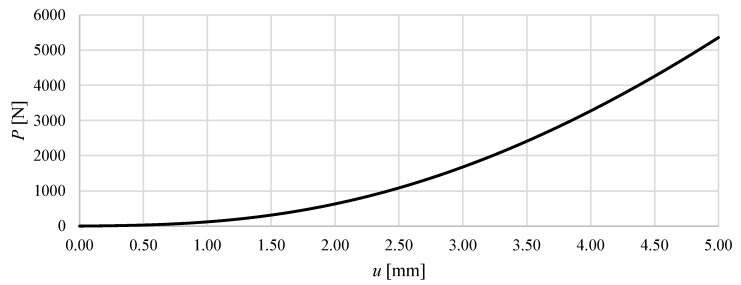
The total vertical reaction *P* as a function of the imposed displacement of the upper nodes of the structure *u* for Model M2, assuming a prestress level of $S_{0}=100$ N.

In the present study, the influence of the level of prestress on structural stiffness and on the load–displacement relationship under prescribed prestress was investigated. Figure 6 and Figure 7 show the total vertical reaction as a function of the imposed displacement. From these graphs, it is evident that Model M1 is significantly stiffer than Model M2. In Model M1, a vertical displacement of 5 mm at the top nodes results in a total reaction force of 69.9 kN, whereas in Model M2 the corresponding reaction is only 5.4 kN—more than an order of magnitude lower. This clearly demonstrates the strong influence of the module configuration on the global stiffness of the structure.

For Model M1, the P(u) relationship is almost perfectly linear. Throughout the displacement range analysed (0–5 mm), the structure exhibits an almost constant stiffness of approximately 13.9 kN/mm, corresponding to the ratio of the total reaction force at u=5 mm. The very high coefficient of determination ($R^{2}\approx 1.000$) for a linear fit confirms the purely elastic and linear nature of the response.

In contrast, Model M2 exhibits a distinct nonlinear behaviour. The initial response is very soft: at u=1.0 mm the reaction force reaches only about 135 N, corresponding to a tangent stiffness of 135 N/mm. As displacement increases, the structure gradually stiffens, with the tangent stiffness increasing nearly tenfold to more than 1.1 kN/mm at u=5.0 mm. This behaviour can therefore be classified as a nonlinear hardening-type response, with increasing stiffness as the deformation progresses. It should be emphasised that the curvature results from tensegrity properties, as the material model used only takes into account linear elastic parameters.

In general, the comparison of the two models highlights fundamental differences in their load transfer mechanisms. Model M1 behaves as a linear elastic system with constant global stiffness, whereas Model M2 shows a nonlinear stiffening response, typical of structures where large initial displacements and geometric effects dominate the early stages of loading, and effective force transfer develops only at larger deformations.

**Figure 8:**
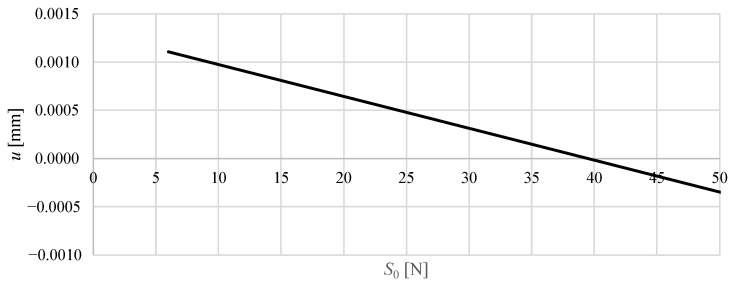
The vertical displacement of selected upper node of the M1 structure as a function of the prestress level $S_{0}$, assuming a constant load of 2.5 N acting on the four upper nodes.

**Figure 9:**
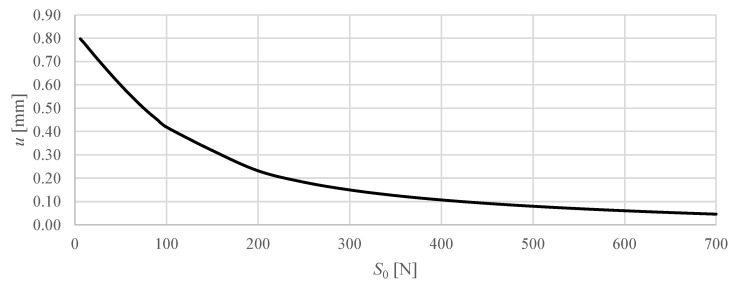
The vertical displacement of selected upper node of the M2 structure as a function of the prestress level $S_{0}$, assuming a constant load of 2.5 N acting on the four upper nodes.

Figure 8 and Figure 9 show the vertical displacement of the upper nodes as a function of the applied prestress force, while keeping the external load constant.

For Model M1, the displacements are extremely small across the entire prestress range. Increasing the prestress from 6 N to 100 N causes the vertical displacement $u_{Z}$ to decrease from about 0.0011 mm to −0.0020 mm. The curve is almost linear and symmetric around zero, indicating that even relatively high prestress levels produce only minor adjustments in the node positions. This reflects the high inherent stiffness of the M1 configuration, where the prestress has a marginal effect on the global deformation under constant load.

In contrast, Model M2 shows a markedly larger response to prestress. With increasing prestress from 6 N to 700 N, the vertical displacement of the top nodes gradually decreases from 0.80 mm to 0.05 mm. The curve exhibits a nonlinear trend with a decrease in slope at higher prestress levels, indicating a progressive stiffening effect: initial increases of $S_{0}$ in prestress produce relatively large reductions in displacement, while further increases of $S_{0}$ yield smaller incremental effects. This behaviour demonstrates that prestressing significantly enhances the stiffness of Model M2 under constant loading, highlighting the role of geometric and module configuration effects in its structural response.

Overall, the comparison clearly shows that prestress has a negligible effect on the already stiff M1 structure, while in the more flexible M2 structure, prestressing is an effective means of controlling deformations and enhancing load-carrying capacity.

The analysis of the eigenvalues of the tensegrity structures was performed in two variants: without prestress ($S_{0}=0$ N); and with prestress ($S_{0}=20$ N). In the case of Model M1, all calculated eigenvalues were positive regardless of the applied prestress (see Table 2), which indicates the complete static stability of the structure. The lowest natural frequency was approximately 12 rad/s and changed only minimally after the introduction of prestress, while the eigenvalues corresponding to higher natural frequencies remained virtually unchanged. The results obtained suggest that the M1 model is characterised by high stiffness and rigid dynamic behaviour, regardless of the level of prestressing.

In the case of the M2 model without prestressing, four zero eigenvalues were observed (see Table 3), corresponding to free displacements occurring despite the correct support of the structure. This behaviour indicates a lack of full static stability and the typical susceptibility of tensegrity structures to displacements in the absence of prestress. After introducing a prestress of $S_{0}=20$ N, the zero modes were raised to small but positive values, which ensured the stability of the structure and eliminated free displacements. The eigenvalues corresponding to the higher eigenmodes remained virtually unchanged, indicating that their dynamic characteristics are less dependent on prestress.

A comparison of both models revealed significant differences in dynamic behaviour. The M1 model is a statically determinate and self-supporting structure, characterised by high stiffness and relatively high natural frequencies even without prestressing. In contrast, the M2 model requires prestressing to achieve full static stability, and its lowest eigenmodes remain low-energy even after prestressing, which may be important in dynamic analysis in the low frequency range. These results highlight the importance of pretensioning in shaping the stiffness and natural vibration characteristics of tensegrity structures.

The observed changes in natural frequencies with prestress arise because the prestress contributes directly to the global stiffness matrix of the tensegrity lattice. By increasing the effective stiffness while the mass distribution remains constant, the ratio of stiffness to mass changes, resulting in higher natural frequencies. This effect is more pronounced for low-frequency modes and in flexible structures, such as M2, where prestress plays a dominant role in stabilising the geometry and enhancing dynamic rigidity.

The role of prestress in the elimination of infinitesimal mechanisms was confirmed by the eigenvalue analysis, which showed that the zero modes observed for $S_{0}=0$ N became small positive values for $S_{0}=20$ N. To complement this observation, global and local stability checks were performed. The global stability of the tensegrity modules was verified by examining the positive definiteness of the global stiffness matrix. In the ABAQUS™ 2019 solver, a loss of positive definiteness manifests itself as a warning of numerical singularity, which was not observed for any of the prestress levels analysed. This confirms that both configurations remained globally stable within the considered load range. Local stability was assessed by comparing the axial compression forces in the struts with the corresponding Euler critical loads, which showed that all members remained below their elastic buckling limits. Imperfections were not introduced because any imprecision in the positions of the nodes disturbs the equilibrium configuration and leads to a divergence of the tensegrity model.

## 4. Discussion

The computational modelling approach presented in this study demonstrates the feasibility of implementing prestressed tensegrity structures as cores in sandwich panels, revealing significant insights into their mechanical behaviour and potential engineering applications. The comparative analysis between the M1 and M2 configurations highlights fundamental differences in structural response that have important implications for practical design considerations.

The implementation of accurate prestress distributions through singular value decomposition and manual force specification represents a significant advancement in tensegrity modelling capabilities. Unlike conventional ABAQUS™ 2019 automatic stress initialisation, the proposed method ensures precise control over the forces of individual members. This precision is critical because tensegrity structures exhibit extreme sensitivity to prestress variations, where small deviations can lead to loss of structural stability or significant changes in global stiffness.

The results of the eigenvalue analysis provide crucial insights into the stability characteristics of different configurations of tensegrity. The consistently positive eigenvalues of the M1 model, independent of the level of the prestress, indicate a statically determinate system with inherent structural stability. This behaviour makes M1 configurations suitable for applications requiring predictable, linear response characteristics and minimal sensitivity to prestress variations. Conversely, the M2 model’s zero eigenvalues in the unstressed state, transitioning to small positive values with prestress application, demonstrate the typical tensegrity behaviour where structural stability emerges from prestress-induced geometric stiffening.

The difference in structural stiffness between the M1 (13.9 kN/mm) and M2 (0.135–1.1 kN/mm variable) configurations reveals the importance of module arrangement and connection topology in determining global behaviour. However, this fundamental difference in behaviour characteristics has critical implications for their application in sandwich panel cores with controllable properties.

The linear elastic response of the M1 configuration and the minimal sensitivity to prestress variations, while advantageous for conventional structural applications, render it unsuitable for sandwich panels requiring controllable mechanical properties. The negligible displacement response to prestress changes indicates that M1 cores cannot provide the adaptive stiffness control necessary for smart sandwich panel systems. The inherently high and constant stiffness of M1 structures eliminates the possibility of real-time property adjustment through prestress manipulation.

In contrast, the M2 model demonstrates ideal characteristics for controllable sandwich panel cores. The substantial sensitivity of the prestress, evidenced by the reduction in displacement from 0.80 to 0.05 mm with an increase in prestress from 6 N to 700 N, provides the foundation for active structural control. The nonlinear hardening behaviour and variable stiffness characteristics of M2 enable precise tuning of panel properties to match specific loading conditions or performance requirements. This adaptability makes M2 configurations particularly suitable for applications requiring responsive structural behaviour, such as morphing aircraft structures, adaptive building systems, or deployable space structures where property control is essential for optimal performance.

The successful implementation of the proposed modelling approach requires careful consideration of manufacturing tolerances and assembly procedures. The high sensitivity of tensegrity structures to geometric imperfections requires precise fabrication methods and quality control procedures. The manual prestress specification method, while computationally accurate, must be translated into practical prestressing procedures that can be implemented during panel assembly.

The modular nature of tensegrity systems offers advantages for scalability and manufacturing customisation. Individual modules can be prefabricated and assembled into larger panel systems, potentially reducing manufacturing complexity and enabling the field assembly of large-scale structures. However, the critical importance of accurate prestress implementation requires careful development of assembly procedures and quality control protocols.

Several limitations of the current study warrant recognition and suggest directions for future research. The integration of tensegrity cores with sandwich panel facesheets requires an investigation of interface bonding methods and load transfer mechanisms that maintain the unique characteristics of both systems. Long-term behaviour considerations, including creep, relaxation, and fatigue effects, are essential for practical applications, but were not addressed in this study. The maintenance requirements of prestresses and their variation over time could significantly impact the practical viability of tensegrity-based sandwich panels in long-term structural applications.

## 5. Conclusions

This study demonstrates a finite element modelling framework for sandwich panel cores based on tensegrity, combining analytical prestress determination with implementation in ABAQUS™ 2019. The main findings can be summarised as follows:

- The proposed prestress specification method based on singular value decomposition ensures precise force control and overcomes limitations of conventional stress initialisation in commercial FEA.
- Comparative analysis of two multi-module configurations revealed fundamentally different behaviours: M1 exhibits high and constant stiffness with minimal sensitivity to prestress, while M2 shows a strong variation in prestress-dependent stiffness.
- The adaptive properties of M2 make it particularly promising for applications in smart sandwich panels, deployable structures, and systems that require a tunable mechanical response, while M1 is more suited for applications demanding high static stiffness and stability.

In general, tensegrity-based cores represent a novel direction for lightweight and adaptive sandwich structures. In this work, an ideal truss model (axial-only) of the tensegrity module was adopted, which allows precise control of prestress and assessment of global stability. Effects such as strut bending stiffness, local end-fixity, and bending in connection zones are not included in the current model; detailed sensitivity analyses with beam elements would require a fundamentally different approach and are therefore planned for future work. Moreover, the present study focusses on short-term static behaviour and stability, while the authors recognise that the long-term performance of prestressed tensegrity cores may be affected by time-dependent mechanisms such as creep, relaxation, and fatigue. These effects can gradually attenuate the prestress and modify the global stiffness and stability of the core. A detailed investigation of these long-term phenomena, supported by experimental verification, will form an important direction for future research. Future studies should also address manufacturing tolerances, facesheet–core interaction, and practical prestressing methods to ensure the viability of tensegrity-based sandwich structures in engineering applications.

## Figures and Tables

**Table 1:** Prestress distribution across struts and cables. The values of prestressing forces resulting from the SVD distribution for a four-strut simplex and the values of initial prestressing of bars are shown, assuming that the struts are made of elements with a circular cross-section with a radius $r_{s}=2.01$ mm, and cables with a circular cross-section with a radius $r_{c}=0.90$ mm. $S_{0}$ is the scale factor, given in N.

	Prestress Force	Stress for $S_{0}=100$ N, [MPa]
Struts	0.103757$\cdot S_{0}$	−2.837957
Top cable	0.146735$\cdot S_{0}$	5.766321
Bottom cable	0.295501$\cdot S_{0}$	4.077392
Middle cable	−0.361090$\cdot S_{0}$	11.612456

**Table 2:** Comparison of eigenvalues and natural frequencies for a structure without prestressing and with prestressing equal $S_{0}=20$ N for Model M1.

Structure Without Prestress ($S_{0}=0$ N)			Structure With Prestress ($S_{0}=20$ N)		
Mode	Eigenvalue	Frequency (rad/s)	Mode	Eigenvalue	Frequency (rad/s)
1	145.23	12.05	1	145.35	12.06
2	145.47	12.06	2	145.59	12.07
3	694.18	26.35	3	694.47	26.35
4	1167.00	34.16	4	1167.20	34.16
5	1480.90	38.48	5	1481.00	38.48
6	1763.10	41.99	6	1763.30	41.99
7	2302.00	47.98	7	2302.40	47.98
8	2326.20	48.23	8	2326.30	48.23
9	3725.50	61.04	9	3725.70	61.04
10	4143.30	64.37	10	4143.50	64.37

**Table 3:** Comparison of eigenvalues and natural frequencies for a structure without prestressing and with prestressing equal $S_{0}=20$ N for Model M2.

Structure Without Prestress ($S_{0}=0$ N)			Structure With Prestress ($S_{0}=20$ N)		
Mode	Eigenvalue	Frequency (rad/s)	Mode	Eigenvalue	Frequency (rad/s)
1	0.00	0.00	1	0.48	0.69
2	0.00	0.00	2	0.83	0.91
3	0.00	0.00	3	1.28	1.13
4	0.00	0.00	4	2.57	1.60
5	993.33	31.52	5	993.80	31.53
6	1729.80	41.59	6	1730.10	41.59
7	2279.10	47.74	7	2280.20	47.75
8	2993.90	54.72	8	2994.60	54.72
9	3398.50	58.30	9	3399.90	58.31
10	5892.40	76.76	10	5893.00	76.77

## Data Availability

The original ABAQUS™ 2019 input files used in the study are openly available in Zenodo at https://doi.org/10.5281/zenodo.17132705.
